# Supplementary material for: Abiotic Stress in Crop Production
Source: Int J Mol Sci. 2023 Apr 1;24(7):6603. doi: 10.3390/ijms24076603 (PMC10095105; doi:10.3390/ijms24076603)
Supplement: Supplementary file 1 [file ijms-24-06603-s001.zip › Supplemetary material S1.pdf]

Supplemetary material S1

Methodology for finding publications focused on abiotic stress

Data included in Panel 1B were derived from Clarivate Web of Science (www.webofscience.com). Data were selected by following criteria:

Topic 1: wheat / sugar beet / maize / potato / barley / sunflower / grapes / rapeseed / tomato / apple

Topic 2: drought / salt / cold / heat

Document type: Article

Categories: Plant Sciences, Agronomy, Environmental Sciences, Agriculture Multidisciplinary, Biochemistry Molecular Biology, Soil Science, Horticulture, Water resources, Genetics Heredity, Biology, Forestry, Cell Biology, Physiology
