# Supplementary material for: Abiotic Stress in Crop Production
Source: Int J Mol Sci. 2023 Apr 1;24(7):6603. doi: 10.3390/ijms24076603 (PMC10095105; doi:10.3390/ijms24076603)

Supplemetary material S2

The methodology of bioinformatics analysis.

The list of genes responsive to abiotic stressors was generated by the Expression Atlas database [203]. Transcriptomic studies were searched for Kingdom: Plants; Experiment type: All; Species: Arabidopsis thaliana; Experimental factors: heat stress/drought stress/cold stress/salinity. Next, only works focused on the seedling developmental stage were manually selected. In total, 11 transcriptional studies were employed in the further analysis (two studies of heat-responsive transcriptome, three studies of cold-responsive transcriptome, three studies of drought-responsive transcriptome, and three studies of salt-responsive transcriptome). A list of differentially expressed genes was generated with criteria: 1) log2-fold change equal or higher than one and 2) adjusted p-value (t-test p-value adjusted using the Benjamini-Hochberg procedure of false discovery rate correction) was set to 0.05. All genes meeting these criteria were used for further analyses. A graphic representation of the analysis is shown in the figure below.

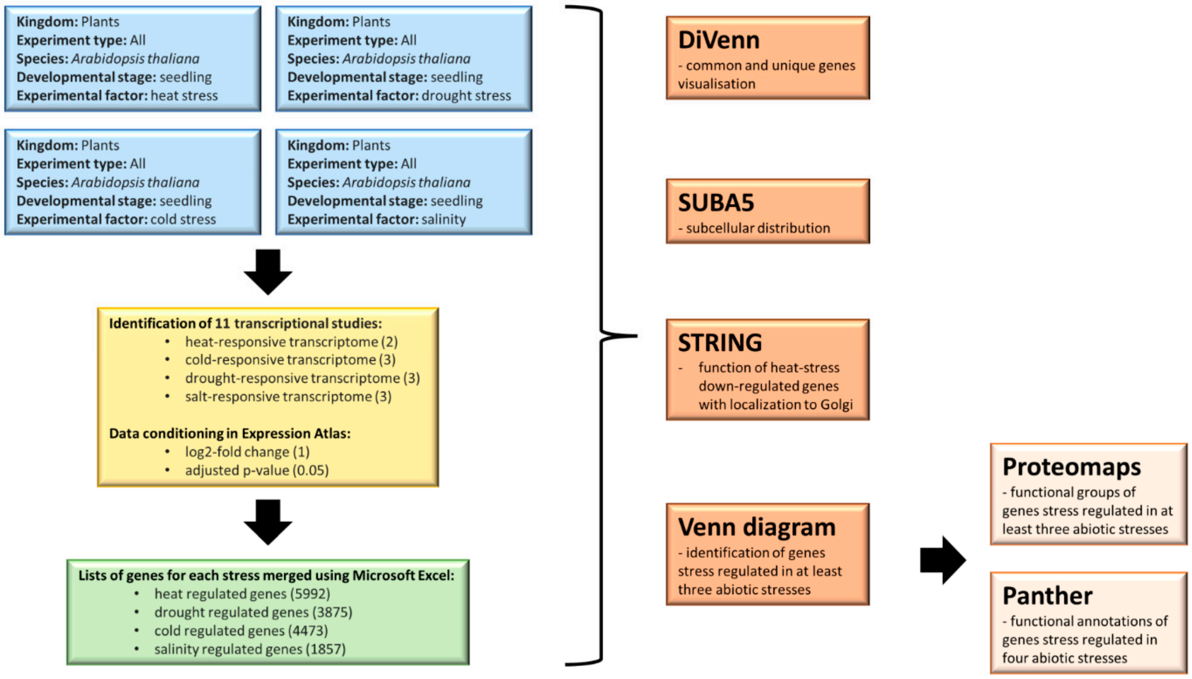

Supplement: Supplementary file 1 [file ijms-24-06603-s001.zip › Supplemetary material S2.pdf]
